# Pseudomembranous necrotizing laryngotracheobronchitis due to *Mycoplasma pneumoniae*: a case report and literature review

**DOI:** 10.1186/s12879-022-07160-5

**Published:** 2022-02-23

**Authors:** Wu Lei, Zhang Fei-Zhou, Chen Jing, Li Shu-Xian, Wu Xi-Ling, Tang Lan-Fang

**Affiliations:** Department of Pulmonology, The Children’s Hospital, Zhejiang University School of Medicine, National Clinical Research Center for Child Health, 3333 Binsheng Rd, Hangzhou, 310052 Zhejiang China

**Keywords:** Pseudomembranous necrotizing laryngotracheobronchitis, *Mycoplasma pneumoniae*, Bronchoscopy, Glucocorticoid, Children

## Abstract

**Background:**

Pseudomembranous necrotizing laryngotracheobronchitis refers to an acute diffuse necrotizing inflammation in the mucosa of the larynx, trachea, and bronchus. It often occurs in infants and children having viral infections secondary to bacterial infections. *Mycoplasma pneumoniae* (*M. pneumoniae*) is a common pathogen that causes pneumonia in children. In recent years, serious complications due to *M. pneumoniae* infection, including necrotizing pneumonia, pulmonary embolism, and pleural effusion, have been increasingly reported.

**Case presentation:**

An 11-year-old girl was admitted to our unit with cough, fever, and hoarseness persistent for a week. The results of the *M. pneumoniae* serological test, PCR examination with bronchial aspirate and bronchoalveolar lavage fluid (BALF), next-generation sequencing (mNGS) for BALF, all suggested the presence of *M. pneumoniae* infection. High-resolution CT scanning of the chest showed inflammation of the middle and lower lobes of the right lung. By bronchoscopy, the necrosis of the vocal cords, trachea, and bronchial mucosa was observed; each bronchial lumen contained a large amount of white viscous sputum. Pathological findings for bronchial mucosa suggested inflammatory necrosis. After administration of azithromycin and glucocorticoids, the symptoms of the patients were ameliorated. After 2 weeks post-discharge, the X-ray scan of her chest indicated the pneumonia resolution in the right lung.

**Conclusions:**

In patients with pneumonia due to *M. pneumoniae* infection, which causes obvious hoarseness, bronchoscopy is necessary even if the lung lesions are not massively consolidated. When necrotizing lesions of the larynx, trachea, and bronchi are detected by bronchoscopy, the necrotic tissues in the corresponding parts should be conducted tissue biopsy for pathological examination. Apart from macrolide antibiotics, the administration of small doses of glucocorticoids is necessary.

## Background

Acute laryngotracheobronchitis can be classified into acute obstructive laryngotracheobronchitis and acute fibrinous laryngotracheobronchitis. The latter, which is rarer than the former, is also called acute pseudomembranous necrotizing laryngotracheobronchitis and is a life-threatening illness [1]. The causes of pseudomembranous necrotizing laryngotracheobronchitis are mostly gram-positive bacterial infections (e.g., *Staphylococcus aureus, Streptococcus hemolyticus*, and *Streptococcus pneumoniae*) [2–4] that are secondary to viral infections (e.g., influenza virus, adenovirus), causing necrotizing damages to the larynx, trachea, and bronchial mucosa. In severe cases, falling off of the pseudomembrane, necrotizing mucus, and phlegm plugs, occur, that block the airway, thereby causing ventilatory dysfunction, eventually leading to breath failure and even death [5, 6].

*Mycoplasma pneumoniae* (*M. pneumoniae*) is a common pathogen responsible for respiratory tract infections in children. These present with various clinical manifestations, ranging from mild upper respiratory tract infections to severe necrotizing pneumonia [7]. Recently, with continuing in-detail studies on *M. pneumoniae*, researchers report that it not only causes severe lung diseases, such as necrotizing pneumonia, pulmonary embolism [8], pulmonary atelectasis [9], plastic bronchitis [10], and bronchiolitis obliterans [11] but also leads to extrapulmonary diseases, including meningoencephalitis [12], hepatitis [13], and pericarditis [14].

Reports on acute pseudomembranous necrotizing laryngotracheobronchitis secondary to *M. pneumoniae* infection are rather rare. To our knowledge, only two cases have been previously reported worldwide [15, 16]. Herein, we report the case of treating a patient with *M. pneumoniae*-related acute pseudomembranous necrotizing tracheobronchitis. Our clinical experience may improve clinicians’ understanding of the disease.

## Case presentation

A previously healthy, 11-year-old girl was admitted to the hospital on September 17, 2021, with main complaints of persistent cough, fever, and hoarseness for a week. She was born at full-term, with vaginal delivery, and weighed 3.75 kg at birth. Her parents had a non-consanguineous marriage. Neither any other associated diseases nor the use of any medicines was gathered.

**Fig. 1:**
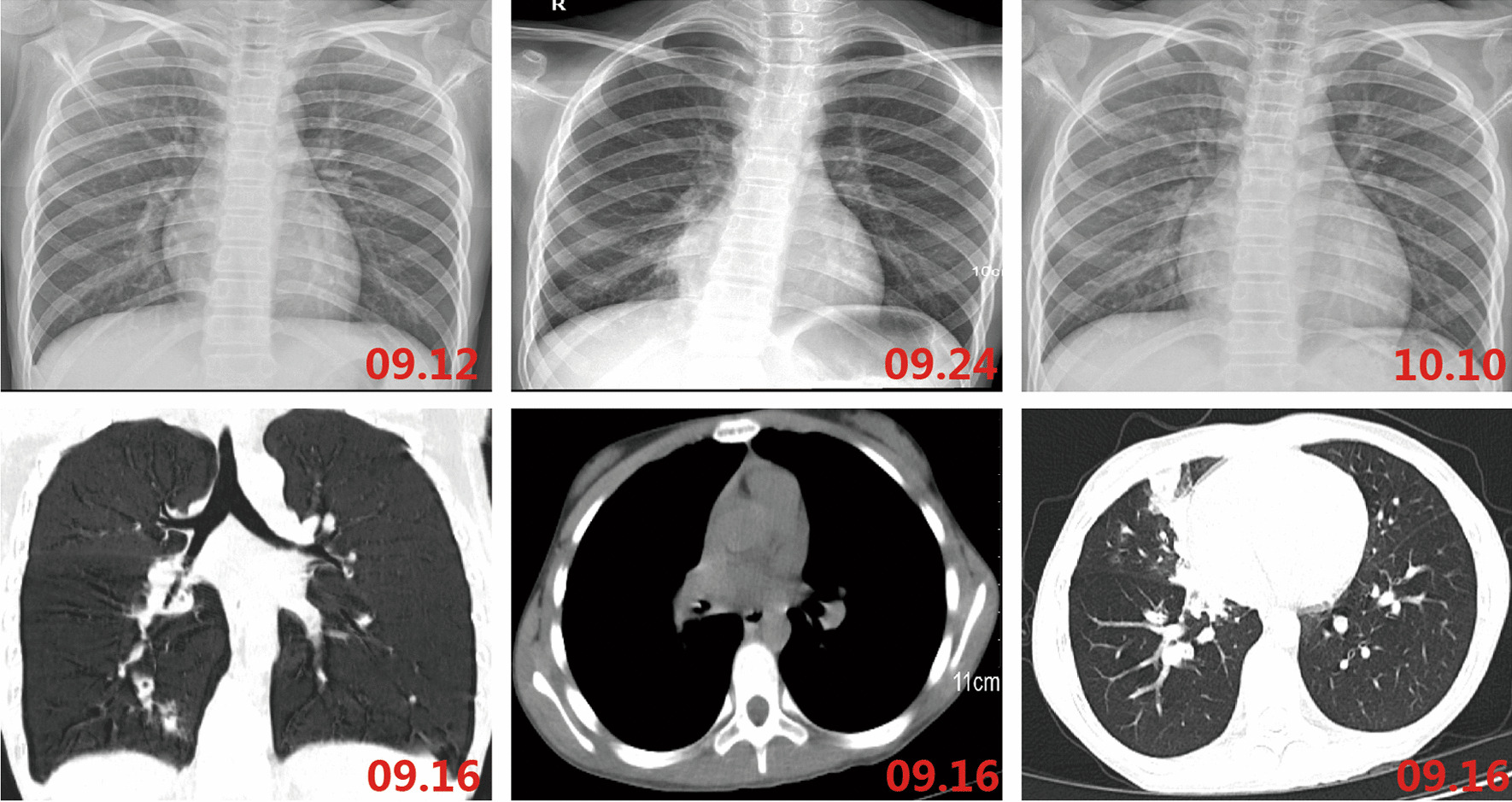
The chest imaging of our case. Chest radiograph showed a fuzzy patchy shadow on the right lower lung. HRCT revealed inflammation of the middle and lower lobe of the right lung. Follow-up chest radiograph prompted basically normal

The child had a persistent cough for a week. It was an irritating dry cough accompanied by a sore throat and hoarseness. There was no nasal congestion, runny nose, shortness of breath, canine barking cough, or dyspnea. She had a fever peaking at 40 degrees Celsius. The temperature decreased gradually to a normal level after oral administration of Ibuprofen, however, it would rise again after 3–4 h. There were no rashes or convulsions. She was referred to the outpatient clinic of our hospital. No obvious abnormalities were observed in the blood routine tests. The antigen tests for respiratory pathogens were also negative. She was diagnosed with “acute upper respiratory tract infection” and prescribed azithromycin tablets, 0.25 g/time, once/day orally for 2 days (Sept. 10–Sept. 11). However, her coughing worsened, manifesting through a marked increase in frequency, and she could not sleep well at night. Routine blood tests were performed in the second outpatient clinic which showed that both white blood cell counts (14.13 × 10^9^/mL) and the high-sensitivity C-reactive protein (23.56 mg/L) levels were elevated. In addition, the chest radiograph showed a fuzzy patchy shadow on the right lower lung on Sept. 12 (Fig. 1A). This was considered a case of “acute bronchopneumonia” and for clearing the infection, Cefotaxime Sodium 3 g/time, once/day for 2 days (Sept. 12–Sept. 13), followed by Ceftriaxone sodium at 2 g/time, once/day for 3 days (Sept. 14–Sept. 16), were administered. Her cough and fever did not show any significant improvement and she returned to our hospital for follow-up visits. A repeated, routine blood test showed that the white blood cell counts were normal (6.82 × 10^9^/mL), but the high-sensitivity C-reactive protein (34.15 mg/L) levels were still high. A High-resolution CT scan of the chest (HRCT) showed inflammation of the middle and lower lobes of the right lung (Fig. 1D–F). She was diagnosed with “pneumonia” and azithromycin 0.28 g/time, once/day was intravenously administered for a day (Sept.16). Her cough improved a little, but the fever was recurrent. For further diagnosis and treatment, she was admitted to our unit with “pneumonia” on Sept. 17.

Physical examinations suggested that the tonsils had a second-degree enlargement and were covered with white secretion. Wet rales were heard from the right lung and there were no obvious abnormalities in the heart, abdomen, skin, and/or the nervous system**.**

Increased levels of high-sensitivity C-reactive protein (49.39 mg/L), alanine aminotransferase (56 U/L), and lactate dehydrogenase (388 U/L) were observed. The results of serological tests for *M. pneumoniae*, PCR examination for bronchial aspirate and BALF, and mNGS of BALF, all suggested the presence of *M. pneumoniae* infection. All results of laboratory tests are listed in Table 1. There were no obvious abnormalities in the electrocardiogram for the heart and the abdominal color Doppler ultrasound.

**Table 1:** The sequence of her laboratory test results as a hospital inpatient

	17 Sept	18 Sept	19 Sept	20 Sept	21 Sept	22 Sept	23 Sept
White blood cell (4–12 × 10^9^/mL)	8.36				9.94		
High-sensitivity C-reactive protein (0–8 mg/L)	49.39				12.67		
D-dimer (< 0.55 mg/L)		3.97			3.96		
Alanine aminotransferase (< 50 U/L)		56			38		
*M. pneumoniae* RNA of bronchial aspirate			Positive				
*M. pneumoniae* RNA of BALF							Positive
Epstein-Barr virus antibody and DNA				Negative			
MP + CP + LG antibody				Negative		Negative	
Tuberculosis infection T cell detection				IgM5.32			
Lactate dehydrogenase (110–295 U/L)		388			258		
Procalcitonin (0–0.46 ng/mL)	0.273						
Erythrocyte sedimentation rate (0-20 mm/h)	50						
Blood culture					Negative		
BALF culture					Negative		
Tuberculosis smear examination of BALF							Negative

**Fig. 2:**
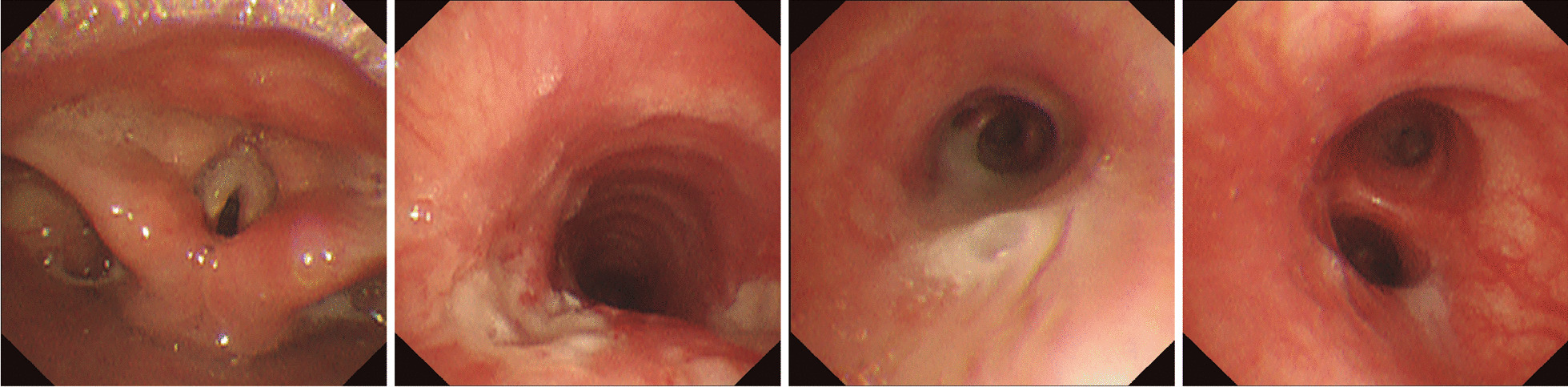
The larynx, trachea, and bronchi under bronchoscopy. Obviously swollen left vocal cords, white strips at the lower edge of the right vocal cords were observed. Moreover, ulcers were observed in the upper and middle trachea, with local depressions, more yellow-white secretions on the surface, and smaller granulation-like tissues

**Fig. 3:**
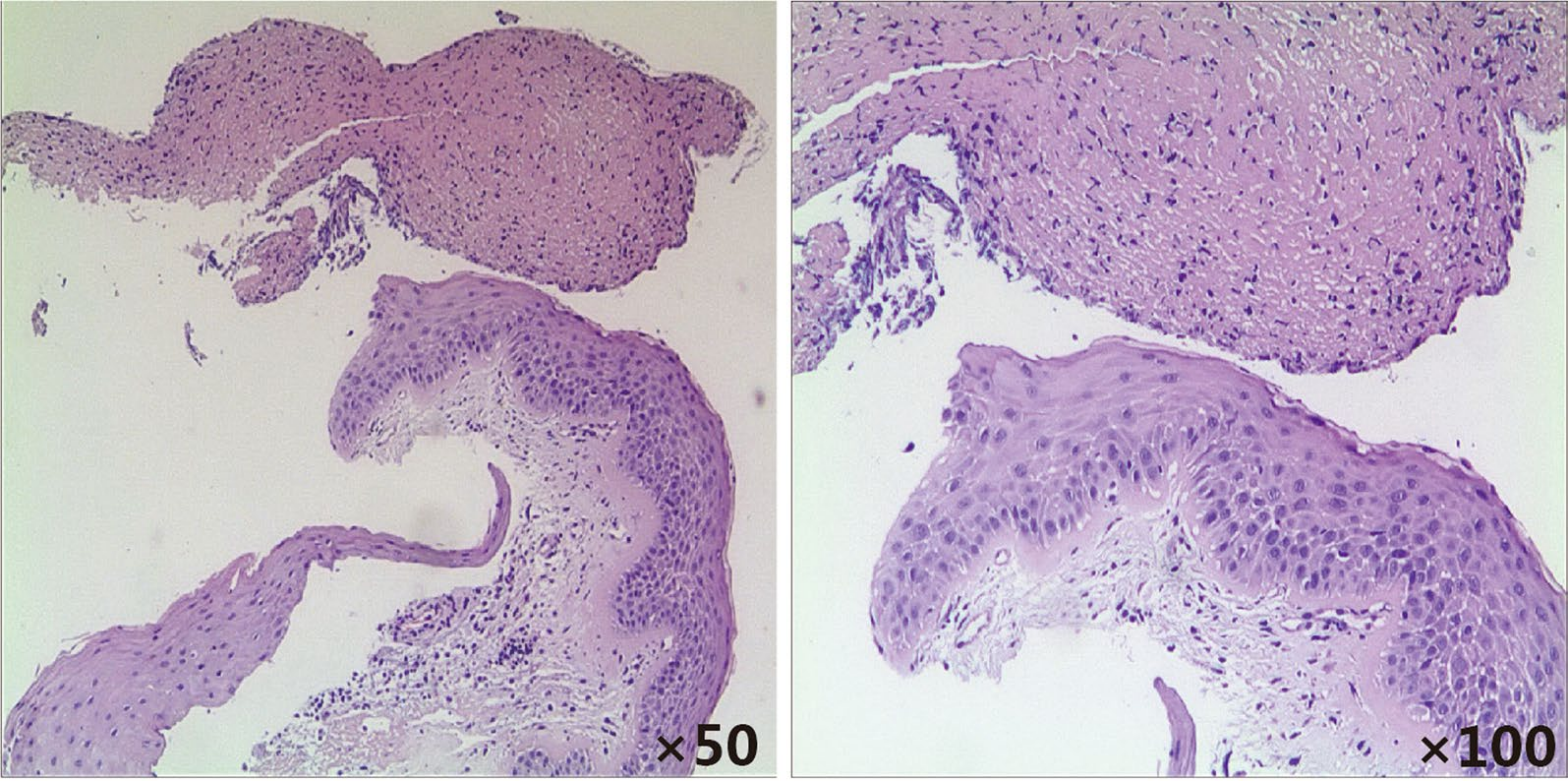
Hematoxylin–eosin staining of clamped tracheal mucosa. Pathology reveals exuding cellulose, chronic mucosal inflammation, and inflammatory necrotic tissues

**Fig. 4:**
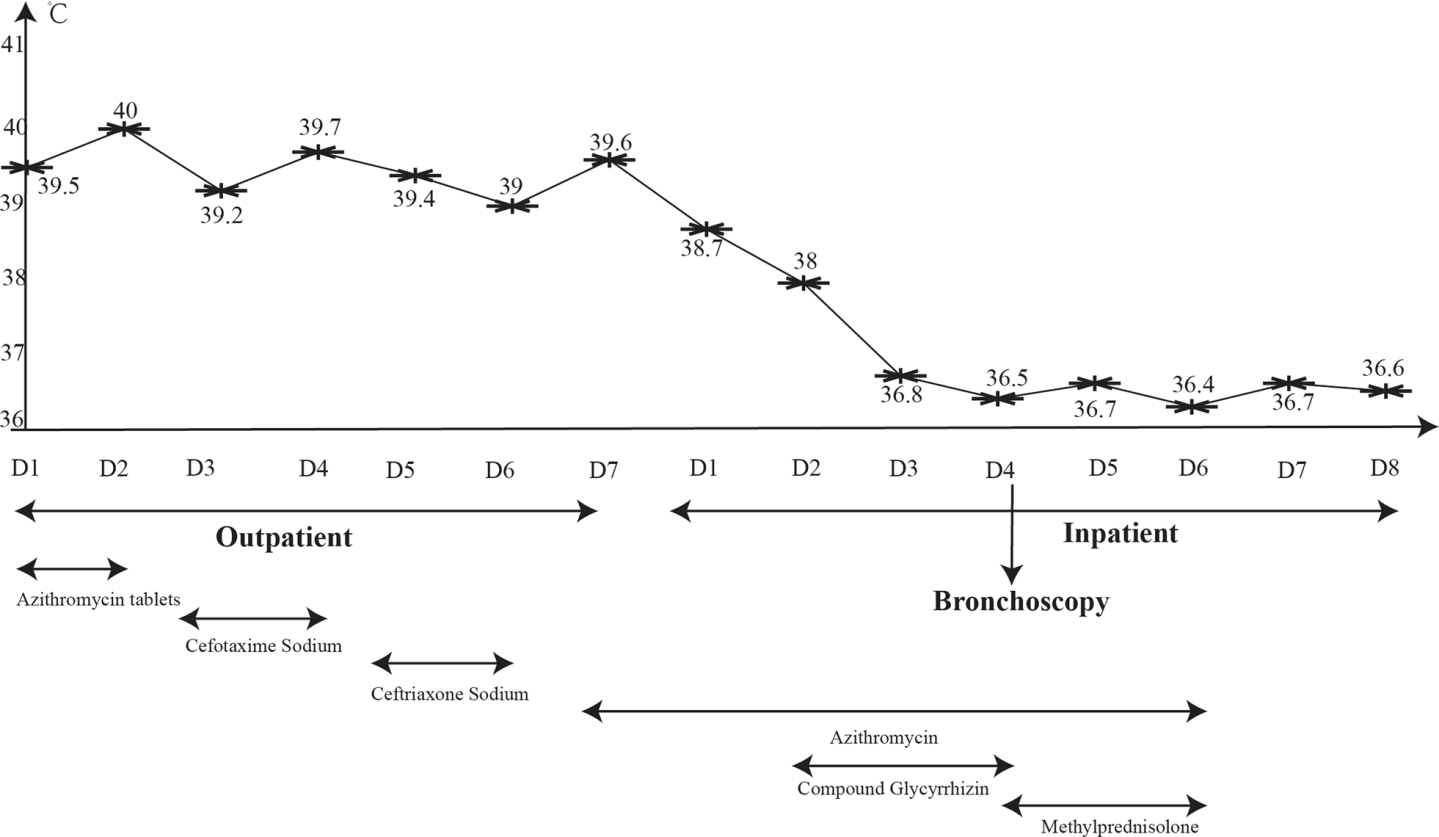
Timeline of the disease process

After admission, azithromycin (Sept.17–Sept. 22) for anti-infection, methylprednisolone (Sept.20–Sept.22) for anti-inflammation, and Compound Glycyrrhizin (Sept.18–Sept.20) for liver protection effects were administered. Bronchoscopy was performed on Sept. 20 and obviously swollen left vocal cords and white stripes at the lower edge of the right vocal cords were observed. Moreover, ulcers were observed in the upper and middle trachea, with local depressions, abundant yellow-white secretions on the surface, and smaller granulation-like tissues. Moreover, in each bronchial lumen, abundant yellow and white viscous secretions were observed (Fig. 2). The pseudomembranes were removed and bronchoalveolar lavage (BAL) was performed. Pathology through the tracheal mucosal biopsy showed cellulose exuding, chronic mucosal inflammation, and inflammatory necrotic tissues (Fig. 3). Finally, the body temperature returned to normal levels on Sept 20, coughing decreased on the fourth day, and hoarseness improved by Sept 22. By Sept 24, her body temperature was normal for continuous 48 h; occasionally coughing but no hoarseness was noted. The chest radiograph demonstrated the presence of right lower lung pneumonia (Fig. 1B). She was discharged (Fig. 4). On Oct. 10, her chest radiograph was found to be back to normal (Fig. 1C).

## Discussion and conclusions

Pneumonia caused by *M. pneumoniae* occurs mainly in adolescents, the elderly, immunocompetent individuals, and older children. The clinical manifestations include an irritating cough [17]. In recent years, the proportion of refractory *M. pneumoniae* pneumonia cases has been on a gradual rise. An 11-year-old girl presented fever and cough and was diagnosed with pneumonia upon imaging. She was positive for IgM and RNA of *M. pneumoniae*. Thus, the diagnosis of *M. pneumoniae* pneumonia was confirmed.

Obvious hoarseness in this patient caught our attention and we decided to perform bronchoscopy and mNGS of the BALF for further confirmation. By bronchoscopy, obvious pseudomembranes were observed in the trachea. The pathological results were in line with our expectations, that is, the presence of necrotizing bronchitis. The mNGS of BALF suggested the presence of *M. pneumoniae* infection only and no other pathogens were detected. The diagnosis of *M. pneumoniae*-related acute pseudomembranous necrotizing laryngotracheobronchitis was confirmed. To our knowledge, only two other cases of *M. pneumoniae-*causing pseudomembranous necrotizing laryngotracheobronchitis have been reported previously (Table 2). Both these patients presented with cough and fever. Notably, case 2 was also that of a child, and in addition to cough and fever, obvious hoarseness was also present. Hence, in patients *with M. pneumoniae* pneumonia, having obvious hoarseness, acute pseudomembranous necrotizing laryngotracheobronchitis should be considered during the differential diagnosis.

**Table 2:** Acute pseudomembranous necrotizing laryngotracheobronchitis associated with *M. pneumoniae* infection

Cases	Sex	Age (years)	Chief complaints	*M. pneumoniae* detection	Treatment	Bronchoscopy times	Admission days	Outcome
1^15^	F	29	Cough, fever, sore throat	PCR + IgM	Moxifloxacin, corticosteroid	1	7	Recover
2^16^	F	2	Cough, hoarseness, dyspnea	mNGS + IgM	Azithromycin, ceftriaxone, methylprednisolone	3	21	Recover
Current case	F	11	Cough, fever, hoarseness	PCR + IgM + mNGS	Azithromycin, methylprednisolone	1	8	Recover

*PCR* polymerase chain reaction, *F* female

As acute pseudomembranous necrotizing laryngotracheobronchitis is relatively rare, the underlying mechanisms remain unclear. Accumulating data show that the initiating factors of acute pseudomembranous necrotizing laryngotracheobronchitis are viral infections. Subsequent bacterial infections are mostly caused by the Gram-positive bacteria (e.g., *Staphylococcus aureus*) [2–4]. Although leukotoxin crucially contributes to *Staphylococcus aureus*-induced necrotizing laryngotracheobronchitis and pneumonia, owing to the destruction of the airway epithelial cells and lungs [18], the mechanisms underlying *M. pneumoniae-*induced necrotizing laryngotracheobronchitis and pneumonia have not yet been reported. These may possibly be related to the direct destruction of the trachea and bronchial mucosa by *M. pneumonia*, the toxic changes in tracheal epithelial cells, and excessive immune responses of the body, similar to the mechanism of action of *M. pneumoniae*-associated necrotizing pneumonia [19].

Although three patients with *M. pneumoniae* infection-related acute pseudomembranous necrotizing laryngotracheobronchitis have been reported to recover, necrotizing tissue, phlegm plugs, and pseudomembrane may fall off and block the airway, thereby causing a critical situation. In patients with *M. pneumoniae* infections, recognizing the complications outside the lung is equally essential in addition to the lung injury. For acute pseudomembranous necrotizing laryngotracheobronchitis, bronchoscopy is a crucial method to diagnose and remove pseudomembranes, thereby alleviating airway obstruction [4, 20]. All three cases of *M. pneumoniae* infection-related acute pseudomembranous necrotizing laryngotracheobronchitis patients have reportedly undergone bronchoscopy, which further confirmed the diagnosis and were treated by the removal of the pseudomembranes and for BAL. In children with *M. pneumoniae* infection and unexplained symptoms (e.g., unrelieved hoarseness, persistent fever), bronchoscopy should be performed for further investigation. Removing the pseudomembranes and washing out the bronchial lumen may improve their condition.

Recently, it has been proposed that the severe inflammatory reactions after *M. pneumoniae* infection are caused mainly by abnormal immune responses. Therefore, in patients who rapidly progress in the acute stage, severe cases, and those with extrapulmonary complications, early application of glucocorticoids should be considered apart from macrolides administration, to quickly relieve symptoms, shorten the course of the disease, and increase the cure rate [21]. As in the two previously reported cases, methylprednisolone (1 mg/kg/day) was administered to our patient on the second day of admission. The fever, cough, and hoarseness of the child were significantly relieved.

In conclusion, in *M. pneumoniae* pneumonia patient with obvious hoarseness, acute pseudomembranous necrotizing laryngotracheobronchitis should be considered in the differential diagnosis; bronchoscopy and BAL should be performed early during the course of treatment. Besides macrolide antibiotics, glucocorticoids should be administered in these refractory cases.

## Data Availability

All data generated or analyzed during this study are included in this published article [and the additional information files].

## Abbreviations

*M. pneumonia*: Mycoplasma pneumonia
HRCT: High-resolution CT
BAL: Bronchoalveolar lavage
BALF: Bronchoalveolar lavage fluid
mNGS: Next-generation sequencing
